# Hearing Loss Prevention Training for Spanish-Speaking Farmworkers: Protocol for a Community-Engaged Hybrid Type 1 Pilot Study

**DOI:** 10.2196/93134

**Published:** 2026-10-05

**Authors:** Laura Coco, Tracy L Finlayson, Nicolas López-Gálvez, Priscila Ruedas, Ana L Rosiles, Briana Toji Ruiz, Brianna Angel, Emma Torres, María Luisa Zúñiga

**Affiliations:** 1 School of Speech, Language, and Hearing Sciences, College of Health and Human Services, San Diego State University, 5500 Campanile Drive, San Diego, CA, 92182, United States, 1 6195943151; 2 School of Public Health, Global Health Management and Policy, College of Health and Human Services, San Diego State University, San Diego, CA, United States; 3 School of Public Health, Environmental Health, College of Health and Human Services, San Diego State University, San Diego, CA, United States; 4 Arizona Center for Rural Health, Tucson, AZ, United States; 5 Lideres Campesinas, El Centro, CA, United States; 6 Campesinos Sin Fronteras, Yuma, AZ, United States; 7 School of Social Work, College of Health and Human Services, San Diego State University, San Diego, CA, United States

**Keywords:** pilot, implementation, community-based participatory research, noise-induced hearing loss, agriculture, farmworkers

## Abstract

**Background:**

Noise-induced hearing loss is permanent yet preventable. Agricultural workers routinely experience hazardous noise exposure, and hearing protection use remains low. Existing interventions emphasize individual knowledge and intentions and have rarely been tested using objective outcome measures. This protocol describes the adaptation and pilot evaluation of an individual-level component of a multilevel noise-induced hearing loss prevention intervention for farmworkers. This pilot focuses on refining and testing a brief digitally delivered novella.

**Objective:**

The objectives of this study are to (1) culturally and linguistically adapt a digitally delivered, Spanish-language entertainment-education hearing protection training (digital novella with facilitated discussion and coached earplug fitting) for farmworkers in the Southwest United States, using community-based participatory research; and (2) pilot test the adapted intervention to assess acceptability, feasibility, and appropriateness and to estimate preliminary effect sizes for changes in objective hearing protector fit.

**Methods:**

This is a single-arm, pre-post, Hybrid Type 1 effectiveness-implementation pilot study. Phase 1 includes formative interviews with farmworkers and farm supervisors (n=30), informed by the Consolidated Framework for Implementation Research, to guide adaptation of training content and delivery. Phase 2 involves a single training session delivered to farmworkers not involved in the formative work (n=15). The primary exploratory outcome is pre-post change in the Personal Attenuation Rating, an objective measure of hearing protector fit. Secondary outcomes include changes in hearing protection beliefs and implementation outcomes (acceptability, feasibility, and appropriateness).

**Results:**

Funding for this study was obtained in July 2025, and the study was registered on ClinicalTrials.gov in December 2025 (NCT07271290). Phase 1 recruitment began in July 2025, and data collection concluded in January 2026, with 30 participants enrolled. Phase 2 pilot recruitment began in July 2026, and 1 participant had been enrolled as of July 2026. Phase 2 data collection is expected to conclude in Spring 2027. Data analysis will follow completion of data collection, and study findings are expected to be published in 2028.

**Conclusions:**

This protocol describes a community-engaged adaptation and pilot evaluation of a culturally tailored, digitally delivered hearing protection microtraining for farmworkers. By pairing objective fit testing with pragmatic implementation measures and systematic adaptation tracking, the study will assess feasibility and estimate preliminary effect sizes to inform a subsequent multilevel trial that integrates supervisor and organizational components.

## Introduction

### Background

Noise-induced hearing loss (NIHL) is permanent yet preventable, and studies indicate that farmworkers are at disproportionate risk of hearing loss due to occupational exposure. Agriculture is among the loudest industries; common tasks (eg, tractors, harvesters, and irrigators) routinely exceed hazardous noise levels (>85 dBA as an 8-h time-weighted average) [1-4]. Chronic exposure to high noise also increases fatigue and stress [5], elevates injury risk [6], and results in disabling hearing loss, with adverse effects on communication, quality of life, depression, and social isolation [4,6-10]. The prevalence of hearing loss in agriculture, forestry, and fishing is 16% nationally [11]. Regional studies have documented even higher rates of hearing loss among farmworkers in the Northeastern (36%) [12] and Midwestern United States (39%) [13]. Our research in the Southwest United States documented hazardous noise exposure during lettuce and vegetable harvesting, a high prevalence of hearing difficulties among farmworkers, and infrequent use of hearing protection despite routine exposure to hazardous noise [14].

Past interventions have primarily targeted individual knowledge, attitudes, and beliefs while giving comparatively less attention to practical earplug-fitting skills, supervisor reinforcement, workplace safety climate, and organizational access to hearing protection, all of which influence hearing protection use. Supervisors shape daily safety practices and are in a position to promote safety culture but are underused in NIHL prevention [15-17]. Many studies rely on intentions or self-reported hearing protection use [18-22], rather than objective outcomes (eg, noise dosimetry, observed hearing protection use). Intentions do not reliably translate to sustained protective behavior [23-25], and workers may underestimate or overestimate hearing protection use [26].

Hearing protection use is influenced by multiple determinants, including knowledge and earplug-fitting skills, confidence in communicating about workplace noise hazards, consistent access to hearing protection, and organizational factors, such as safety climate and supervisory reinforcement [15,16,27,28]. These findings suggest that interventions addressing multiple levels of influence may be more effective than those focusing on knowledge alone.

There is also a striking evidence gap for Latino farmworkers and farmworkers with non-English language preference who comprise the majority of the US agricultural workforce. Many studies fail to report ethnicity or language [21,29] or exclude farmworkers who do not speak or read English [20,27,30,31]. This limitation severely impacts the extent to which findings are generalizable and scalable; in California, only 15% of farmworkers can read English [32,33]. These structural and linguistic barriers underscore the need for training modalities that are portable, low-literacy, and not dependent on written materials or sustained in-person instruction. Representative research is particularly important given documented disparities in hearing protection use by ethnicity [34,35].

Despite the availability of effective hearing protectors, consistent use among farmworkers remains low [14,18,27]. Reported barriers include discomfort, interference with communication, limited access to hearing protection, inadequate training in proper earplug insertion, competing work demands, and workplace norms that do not consistently support hearing conservation [16,18,28,36]. Existing hearing conservation interventions have demonstrated improvements in knowledge and attitudes but have produced mixed effects on hearing protection behaviors, particularly when evaluated using objective measures [18,19,21,28]. These findings highlight the need for interventions that address both individual skills and the broader implementation context in which hearing protection decisions occur.

### Existing Hearing Conservation Interventions and the Need for Adaptation

In the United States, Spanish-language novellas have been used as an entertainment-education strategy to promote health behaviors [37], including encouraging the use of hearing protection [18]. Grounded in social cognitive theory [38,39], the novella approach leverages modeled characters to provide vicarious learning, reinforcing desired behaviors and discouraging harmful ones. When digitally delivered, novellas offer additional advantages for agricultural settings, including standardized content, portability across community and worksite locations, the potential for asynchronous dissemination via mobile platforms, and delivery through audio rather than text, reducing reliance on reading.

Although several hearing conservation interventions have been developed for agricultural populations, few have been designed specifically for Spanish-speaking farmworkers, have incorporated community-engaged adaptation, have evaluated implementation outcomes, or have used objective measures of hearing protector fit [16,28]. Consequently, there remains a need for interventions that are culturally and linguistically appropriate, feasible to deliver in agricultural settings, and designed with future implementation in mind.

Our preliminary discussions with farmworkers and community partners suggested several barriers to noise exposure prevention, including inconsistent access to earplugs and limited understanding of workplace noise hazards. Community partners also identified a need for practical approaches that could be delivered in Spanish and integrated into existing safety training. *Más Vale Usar Protección Auditiva* (It’s Worth It to Use Hearing Protection) is one of the few Spanish-language interventions developed specifically for Spanish-speaking farmworkers [18,40]. It is a brief (~6 min) digital novella that uses modeled characters and culturally relevant narratives to demonstrate hearing protection behaviors and address common misconceptions regarding hearing loss prevention. However, *Más Vale Usar Protección Auditiva* was initially created without feedback from farmworkers or farm managers and has not previously been adapted for the cultural, linguistic, and occupational context of farmworkers in the Southwest United States, creating a need for contextual adaptation.

To support intervention adaptation and future implementation, this study integrates complementary community-engaged and implementation science frameworks. Community-based participatory research (CBPR) guides collaboration with farmworker-serving organizations throughout intervention development, while implementation science frameworks inform adaptation, evaluation, and reporting. Together, these frameworks support the development of an intervention that is evidence-informed, contextually appropriate, and designed for future implementation. Figure 1 and Table 1 summarize the frameworks and their roles within the study.

**Figure 1.**
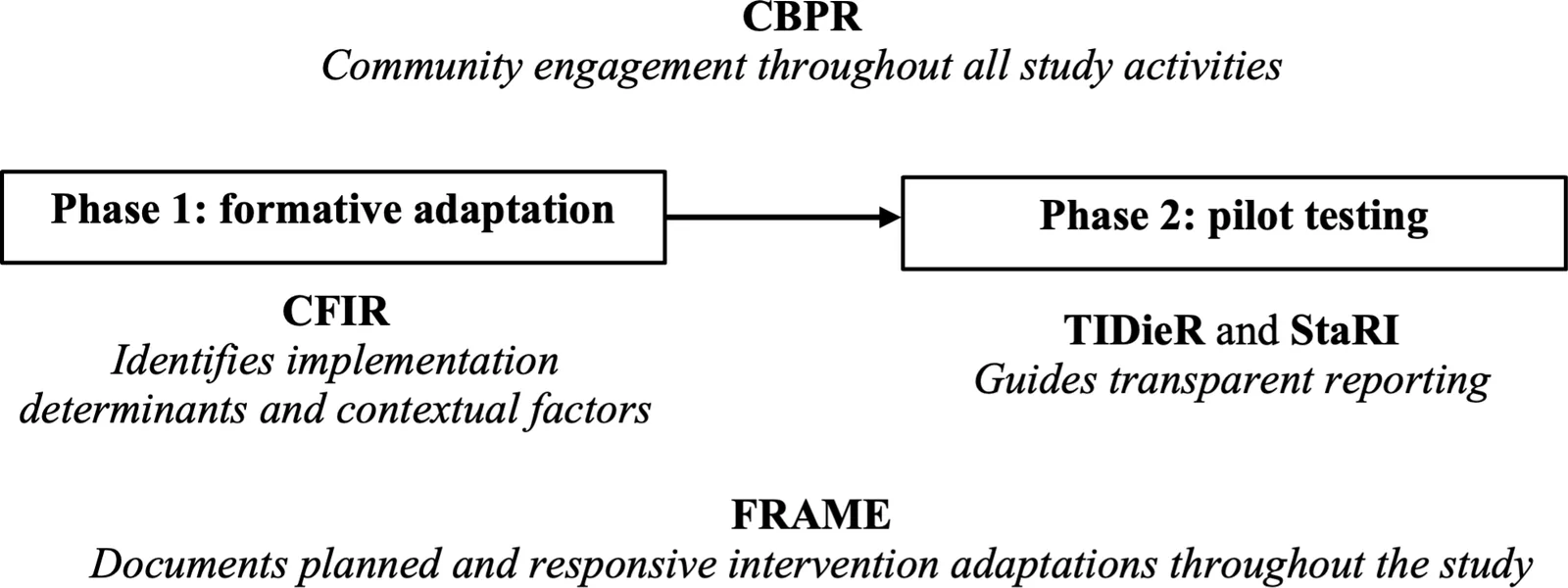
Frameworks guiding adaptation, pilot implementation, and reporting of *Más Vale Usar Protección Auditiva*. Community-Based Participatory Research (CBPR) provides the community-engaged foundation for all study activities. During phase 1, the Consolidated Framework for Implementation Research (CFIR) guides the identification of implementation determinants and contextual factors. The Framework for Reporting Adaptations and Modifications to Evidence-Based Interventions (FRAME) documents planned and unplanned adaptations throughout the study. During phase 2, intervention and implementation reporting follow the TIDieR (Template for Intervention Description and Replication) and StaRI (Standards for Reporting Implementation Studies).

**Table 1.** Frameworks guiding adaptation and implementation activities in the pilot study.

Framework	Function
CBPR^a^	Guides community engagement, co-design, and shared governance
CFIR^b^	Identifies implementation determinants and contextual factors
FRAME^c^	Documents planned and unplanned adaptations
TIDieR^d^	Guides intervention description and replication
StaRI^e^	Guides implementation reporting

a CBPR: Community-Based Participatory Research [41,42].

b CFIR: Consolidated Framework for Implementation Research [43].

c FRAME: Framework for Reporting Adaptations and Modifications to Evidence-Based Interventions [44].

d TIDieR: Template for Intervention Description and Replication [45].

e StaRI: Standards for Reporting Implementation Studies [46].

### Study Objectives

The aims of this study are to (1) adapt an existing digital novella for farmworkers in the Southwest United States and (2) pilot test the adapted training to assess acceptability, feasibility, appropriateness, and preliminary estimates of effectiveness.

## Methods

### Study Design

This study uses a sequential mixed methods Hybrid Type 1 effectiveness-implementation design to adapt and evaluate *Más Vale Usar Protección Auditiva*. Hybrid Type 1 designs primarily evaluate intervention effectiveness while simultaneously collecting implementation data to inform future scale-up and dissemination.

The study consists of 2 sequential phases. Phase 1 includes approximately 30 participants (20 farmworkers and 10 supervisors/managers) and uses qualitative methods to adapt the intervention and identify implementation considerations. Findings from phase 1 will inform modifications to the intervention content, delivery, and implementation procedures. Phase 2 includes approximately 15 farmworkers who did not participate in phase 1 and pilot tests the adapted intervention using a single-arm pre-post design to assess implementation outcomes and estimate preliminary effects on objective hearing protector fit.

The phase 1 sample size was selected to obtain diverse perspectives regarding hearing protection practices, barriers, facilitators, and implementation considerations while supporting intervention adaptation. The phase 2 sample size of approximately 15 participants was selected pragmatically based on anticipated recruitment capacity and available resources and is consistent with recommendations for pilot studies. The sample is intended to assess implementation outcomes, evaluate study procedures, and estimate the magnitude and variability of paired pre-post changes and preliminary effect sizes rather than provide a definitive test of intervention effectiveness. Findings will be used to refine study procedures and inform the design and sample-size calculations of a future fully powered trial.

The primary exploratory outcome assessed in this pilot is the change in Personal Attenuation Rating (PAR), an objective measure of hearing protector fit. Secondary effectiveness outcomes include changes in hearing protection beliefs and the implementation outcomes (acceptability, feasibility, and appropriateness). Additional feasibility data regarding implementation of PAR fit-testing in community-based agricultural settings—including testing time, participant burden, equipment performance, facilitator observations, and logistical challenges—will be collected to inform future community-based implementation.

### Conceptual Frameworks and Reporting Standards

CBPR principles guide collaboration with community partners throughout intervention adaptation and implementation. During phase 1, the Consolidated Framework for Implementation Research (CFIR) informs the identification of implementation determinants and contextual factors influencing intervention delivery, uptake, and sustainability. The Framework for Reporting Adaptations and Modifications to Evidence-Based Interventions (FRAME) is used to document planned and unplanned adaptations and their rationale throughout the study. Intervention reporting follows the TIDieR (Template for Intervention Description and Replication), and implementation reporting follows the StaRI (Standards for Reporting Implementation Studies). Together, these frameworks provide complementary guidance for intervention development, implementation, adaptation, and transparent reporting. See Checklist 1 and Checklist 2 for completed TIDieR and StaRI checklists.

### Intervention Logic and Delivery

Within the broader multilevel intervention, the worker-level module is the intervention evaluated in this pilot study. The intervention consists of a culturally relevant narrative delivered as a digital novella, a facilitated discussion, and hands-on earplug fitting with teach-back coaching. These core components will remain consistent because they are intended to deliver the intervention’s core behavioral and educational functions. Adaptable elements include language, visuals, examples, contextual references, dissemination channels, and delivery logistics to meet local needs. Adaptations will be documented using the FRAME while preserving the intervention’s core functions.

In this pilot study, implementation will be supported through standardized training, a facilitator guide, intervention checklists, and documentation of context-specific adaptations using the FRAME. The intervention will be delivered by trained promotors to evaluate its feasibility and acceptability in this setting.

Together, these components target empirically identified individual-level and interpersonal-level determinants of hearing protection use, including limited fitting skills, low confidence communicating about noise hazards, inconsistent access to hearing protection, and variable safety climate and supervisory reinforcement. By addressing these determinants, the intervention is expected to improve hearing protection knowledge, self-efficacy, and correct earplug use while also supporting positive implementation outcomes, including acceptability, feasibility, and appropriateness. Fidelity of intervention delivery will be assessed through adherence to the core intervention components, including delivery of the digital novella, facilitated discussion, and earplug fitting procedures. Findings from this pilot will inform refinement of the worker-level intervention and future implementation of the broader multilevel intervention integrating worker-, supervisor-, and organizational-level components.

### Setting

The study will be conducted in Imperial County, California, a rural, agriculturally intensive region on the US-Mexico border that shares strong social ties and an interconnected labor market with Mexicali, Baja California. Imperial County is geographically large and sparsely populated, with an estimated 2024 population of 181,724 residents [47]. The majority of residents identify as Hispanic or Latino (86.6%), and 75.3% of persons aged 5 years and older report speaking a language other than English at home [47]. Agriculture is a primary economic driver in the county, supporting thousands of jobs and extensive agricultural production. Imperial County contains approximately 500,000 farmable acres and produces more than 65 crops, including substantial winter vegetable production [47,48].

### Community Partnership and CBPR Procedures

This study follows a CBPR approach to ensure shared governance, transparent decision-making, and sustained capacity building [49,50]. Members of Líderes Campesinas Imperial County serve as coinvestigators who share responsibility for protocol decisions (eg, recruitment strategy, consent language, and adaptation content). A Community Advisory Board (CAB) comprising 7 members (4 representatives from farmworker advocacy groups, 2 public health researchers, and 1 local public-health practitioner) will meet quarterly and on an ad hoc basis as needed. CAB members and partner staff receive honoraria for meetings and time spent on study activities and are offered training in basic research methods (eg, consent procedures, data quality, and qualitative coding) to support local research capacity. All co-design outputs (storyboards, scripts, and fidelity checklists) will be developed iteratively with community coleads; versions and rationale for changes are logged in FRAME and archived in the project audit trail.

Major decisions regarding recruitment procedures, intervention adaptation priorities, implementation strategies, interpretation of findings, and dissemination activities will be reviewed collaboratively by academic and community partners through CAB and project planning meetings. Community partners will participate in reviewing formative findings, prioritizing intervention modifications, and determining whether proposed adaptations are appropriate and feasible for implementation within local agricultural settings. Decisions will be made through discussion and consensus whenever feasible, with adaptation decisions documented in project records and FRAME logs. Community partners will also contribute to the interpretation of study findings and the development of dissemination materials for both community and the scientific audiences.

### Iterative Co-Design and Adaptation Procedures

The research team will work in close partnership with community coleads and the CAB and, using an iterative co-design process, adapt the digital novella and associated facilitation materials for the local agricultural context. Semistructured formative interviews will be used to identify salient noise-exposure contexts, practical barriers to the use of hearing protection, and social and organizational dynamics influencing behavior. Findings from each iteration will inform revisions to narrative content, visuals, language, and delivery logistics, resulting in a field-ready version of the digital novella, facilitation guide, and fidelity checklist for pilot testing. All adaptations, whether planned or responsive, will be systematically documented using FRAME. Adaptations will be documented by the principal investigator (LC) following transcription and analysis of formative interviews, co-design meetings, and pilot implementation activities. Adaptations will be classified according to FRAME dimensions, including timing, decision-maker, level of delivery, nature of the modification, fidelity implications, and rationale.

### Linguistic and Cultural Concordance

All instruments and materials will be developed in Spanish first and then translated or adapted for English as needed. We will use a team-based translation approach with community verification for contextual accuracy, adopt plain-language standards at or below an eighth-grade readability level, and incorporate regionally used terms for hearing protection, such as *tapones* (earplugs) and *protección auditiva* (hearing protection). We will use the Spanish-language items from prior farmworker research; any minor local wording adaptations will be verified with community partners and documented in FRAME.

### Participants and Recruitment

Participants will be recruited through established community partnerships, including members of Líderes Campesinas and other local agricultural worker networks. When possible, purposive sampling will be used to recruit participants with diverse experiences relevant to the study objectives, including variation in agricultural job tasks, years of farm work experience, gender, employer type, supervisory role (for phase 1 supervisors), and prior experience using hearing protection. This approach is intended to capture a broad range of perspectives on noise exposure, hearing protection practices, and implementation considerations. Recruitment materials will be available in Spanish and English and will be distributed through community events, partner organizations, and word-of-mouth referrals. Eligible participants who complete study activities will receive a gift card in recognition of their time and contributions.

### Digital Novella and Adaptation Process

#### Original Digital Novella

*Más Vale Usar Protección Auditiva* (“It’s Worth It to Use Hearing Protection”) is a Spanish-language digital novella that models common barriers and solutions related to hearing protection use through short, dramatized scenes illustrating the consequences of NIHL. For this study, the novella serves as the core didactic component to be culturally and contextually adapted through phase 1.

#### Phase 1: Adaptation

We will use semistructured interviews with farmworkers and supervisors to determine content, format, and delivery changes needed for the novella-based training and its facilitation. Trained bilingual interviewers from Líderes Campesinas will conduct individual interviews lasting approximately 20 to 30 minutes at community locations or other participant-preferred venues. After providing verbal and written informed consent, farmworker participants will view the original “Más Vale” digital novella on a tablet or other device and respond to open-ended prompts that cover: prior experience with hearing protection; reactions to the video (clarity, relevance, what was learned, what was confusing, and what to change); social norms and likely peer responses; practical barriers and facilitators to using hearing protection (eg, pain, time pressure, lack of supply, comfort, availability, and cost); and preferences for dissemination (eg, WhatsApp vs break-time group viewing). All interview prompts will be read aloud by the interviewer to support participants with low literacy. Supervisors will also view the novella and respond to questions that address organizational practices, current provision of hearing protection, fit with existing safety routines, roles and responsibilities for reinforcing use, and practical options for integrating the video into routine safety meetings.

All interviews will be audio-recorded, transcribed verbatim in the original language (deidentified omitting potential identifiers), and translated into English as needed. Coding will map to CFIR domains (intervention characteristics; inner setting such as policies and safety climate; outer setting such as regulations and resources; characteristics of individuals; and process), with inductive codes added based on the data. Two bilingual coders will independently code an initial subset of farmworker and supervisor transcripts, reconcile discrepancies by consensus, and finalize a codebook before coding the remaining interviews. Emergent findings that suggest immediate, low-risk improvements (eg, clarifying an image, simplifying an instruction) will be brought to community partners for early incorporation. Intercoder agreement will be monitored following recommended procedures for qualitative reliability [51].

Analysis will use a rapid qualitative analysis approach informed by the CFIR. Interview summaries will be entered into structured matrix templates organized by CFIR domains and study objectives. The analytic team will review summaries iteratively to identify barriers, facilitators, adaptation needs, and implementation considerations relevant to the intervention. Matrix-based synthesis will be used to compare findings across participant groups (farmworkers and supervisors) and identify patterns that inform adaptation of intervention content, delivery, and implementation strategies. Rapid qualitative methods were selected to facilitate timely integration of findings into the intervention adaptation process while maintaining analytic rigor.

To enhance trustworthiness, multiple analysts will review interview summaries and matrices, with discrepancies discussed by consensus. Analytic decisions, adaptation recommendations, and rationale for changes will be documented through audit trails and FRAME logs. Community partners and members of the CAB will contribute to the interpretation of findings to enhance contextual validity and ensure that emerging themes accurately reflect local experiences and priorities. Detailed documentation of the study setting, participant characteristics, and adaptation process will support the assessment of transferability to other agricultural settings.

Following the analysis, CBPR-informed co-design will be used to revise the script, storyboard, and facilitation materials. Each change will be logged using FRAME, noting when and how the change occurred, whether it was planned or reactive, who made the decision, what was modified (content, context, delivery), the level of delivery, the nature of the change, fidelity implications, and the rationale. Informal cognitive interviews with community members will be used to assess comprehension and cultural resonance of key revisions before finalizing the pilot version.

#### Phase 2: Pilot Delivery

We will evaluate a single-session training of approximately 60 minutes for farmworkers who did not participate in phase 1. After providing consent, participants will complete the baseline outcome measures described below. The facilitator will then deliver the revised novella and a brief hands-on earplug fitting with teach-back coaching. Immediately after the session, outcome measures will be repeated. Following completion of the quantitative outcome measures, participants will complete a brief semistructured exit interview exploring their experiences with the training, perceived barriers and facilitators to hearing protection use, suggestions for improving the intervention, and recommendations for implementation in agricultural workplaces. Fidelity and quality assurance will be tracked with a facilitator checklist that documents delivery of core components (video viewed end-to-end, discussion prompts covered, earplug coaching completed, teach-back achieved), session duration, attendance, and any deviations. Facilitators will be trained with standardized scripts, step-by-step PAR procedures, and mock sessions observed by the principal investigator or designee. Refresher sessions will be conducted if needed. Data quality procedures include bilingual administration, interrater agreement checks on qualitative coding, calibration logs for the fit-testing device, and a contemporaneous audit trail of adaptation decisions. Prior to independent delivery, facilitators will demonstrate competency through observed mock sessions and completion of standardized PAR testing procedures.

### Eligibility

Phase 1 will recruit approximately 20 farmworkers and 10 supervisors/managers. Phase 2 will recruit approximately 15 farmworkers who did not participate in phase 1. Eligibility for farmworker participants includes the following: aged 18 years or older, Spanish- or English-speaking, and currently employed in agricultural work involving routine exposure to powered equipment (eg, tractors, harvesters, irrigation pumps, or similar machinery) or other tasks with potentially hazardous occupational noise exposure. Supervisor participants must be aged 18 years or older, Spanish- or English-speaking, employed in a supervisory or managerial role within an agricultural setting, and able to provide informed consent. Exclusion criteria include medical or ear conditions that preclude the safe use of foam earplugs (eg, acute ear infection or recent ear surgery) or inability to complete study procedures because of cognitive impairment, an inability to follow study instructions, or an inability to complete PAR fit testing despite repeated instruction.

### Outcomes and Measures

For phase 1, the primary outcome is qualitative data from semistructured interviews. Interview guides were drafted by the academic team to align with CFIR constructs and were revised through one round of CAB review and two rounds with Líderes Campesinas partners (see Multimedia Appendix 1). This process produced clearer questions, eliminated redundancy, and added a brief observed earplug-insertion task before and after the novella to understand practical barriers and visual needs for fitting instructions. The observed earplug insertion task involves asking participants to insert foam earplugs as they typically would, followed by the facilitator’s documentation of technique, depth of insertion, and common errors to inform content revisions.

For phase 2, the primary exploratory quantitative outcome is pre-post change in PAR, which will be used to estimate preliminary effect sizes. Participants will undergo fit testing before training to establish a baseline PAR and will be retested immediately afterward using the wireless automated hearing test system (WAHTS; Creare LLC, Hanover, NH, USA) with Audhere software (Creare LLC, Hanover, NH, US; release dated July 15, 2026). Fit testing will be performed using 3M E-A-R Classic disposable foam earplugs (3M; Noise Reduction Rating, 29 dB) following the WAHTS hearing protector fit-testing protocol. The protocol measures unoccluded and occluded hearing thresholds at 500, 1000, 2000, 3000, 4000, 6000, and 8000 Hz to calculate the overall binaural PAR. The system will be calibrated according to the manufacturer’s recommendations before each testing session. Testing will be conducted in a quiet indoor environment that meets the manufacturer’s maximum permissible ambient-noise requirements (60 dBA or 73 dBC). Measurements that do not converge or otherwise fail quality checks will be repeated according to the WAHTS protocol.

The secondary effectiveness outcome is the change in hearing protection beliefs, measured with a 14-item Hearing Protection Beliefs Scale that has been previously used with Spanish-speaking farmworkers. Items are rated on a 4-point Likert scale (1=disagree to 4=strongly agree) and summed after reverse-coding negatively worded items; total scores range from 14 to 56, with higher scores indicating more favorable and accurate beliefs about hearing protection. The Spanish-language version previously used with Spanish-speaking farmworkers will be administered. Minor wording modifications identified during the phase 1 adaptation process to improve regional comprehension will be documented but will not alter the underlying constructs or scoring procedures. The scale has previously been used in studies of hearing loss prevention and hearing protection behaviors, including among Spanish-speaking populations [18,23,27,36]. Negatively worded items include statements related to missing warning sounds, interference with work performance, and perceptions that hearing protection is not worth the effort. Internal consistency (Cronbach α) will also be reported descriptively for this sample. This pilot study is not intended to validate the instrument or evaluate its psychometric properties.

Implementation outcomes will be measured using the Acceptability of Intervention Measure, Feasibility of Intervention Measure, and Intervention Appropriateness Measure, 3 brief implementation outcome measures with demonstrated reliability, structural validity, and test-retest reliability in implementation research settings [52]. Each measure consists of 4 items rated on a 5-point Likert scale (1=completely disagree to 5=completely agree). Item responses will be averaged to generate a mean score for each construct, with higher scores indicating greater perceived acceptability, feasibility, or appropriateness of the intervention. Implementation outcomes will be assessed immediately posttraining. Internal consistency will be assessed descriptively in this sample. Quantitative measures will be supplemented by CFIR-guided open-ended questions to explore participants’ perceptions of the intervention and implementation context.

Participants will also report access to hearing protection (eg, employer-provided foam earplugs, reusable earplugs, earmuffs, self-purchased devices, or no access) to characterize workplace resources available at baseline. During the training session, all participants will receive foam earplugs to ensure short-term access to hearing protection.

### Data Analysis

For aim 1, analysis will use a rapid qualitative approach informed by the CFIR Version 2.0 [53-55]. Individual interview transcripts will be used to prepare structured interview summaries, which will serve as the primary analytic unit for matrix-based analysis. Matrix templates will be organized according to the five CFIR domains and study objectives. Interview guides were developed to map to selected CFIR constructs relevant to intervention adaptation and implementation while allowing participants to introduce issues beyond the framework. The analytic team will iteratively review interview summaries to identify barriers, facilitators, adaptation needs, and implementation considerations. Matrix-based synthesis will be used to compare findings across participant groups (farmworkers and supervisors) and identify patterns that inform adaptation of intervention content, delivery, and implementation strategies. Rapid qualitative methods were selected to facilitate timely integration of findings into the intervention adaptation process while maintaining analytic rigor. Inductive codes will be created for themes not adequately captured by CFIR to ensure that novel or context-specific findings are retained.

For phase 2, participant characteristics and implementation outcomes will be summarized descriptively using means and SDs. Paired pre-post changes in PAR and hearing protection beliefs will be summarized using change scores with 95% CIs. Given the small pilot sample and potential nonnormality, Wilcoxon signed-rank tests will be conducted as exploratory analyses. Preliminary standardized effect sizes (Hedges g) will be calculated for paired pre-post changes and interpreted descriptively to estimate the magnitude and variability of intervention effects for planning a future fully powered trial, rather than to provide a definitive test of intervention effectiveness. Mean scores, SDs, and score distributions will be reported descriptively for the acceptability of intervention measure, feasibility of intervention measure, and intervention appropriateness measure. Internal consistency will also be reported descriptively.

Given the brief, single-session design, minimal missing data are anticipated. Analyses will be conducted using available data for each outcome. Participants with missing pre or posttraining PAR measurements will be excluded from analyses involving PAR, whereas participants with missing survey responses will be included in analyses of outcomes for which data are available. Because of the small pilot sample, no imputation procedures are planned. The extent and nature of missing data will be reported descriptively.

Because this is an exploratory pilot study designed to assess feasibility, implementation outcomes, and preliminary signals of effectiveness, no formal adjustment for multiple comparisons will be applied. Results will be interpreted cautiously and used to inform future hypothesis-driven studies rather than to provide definitive tests of intervention effectiveness.

Quantitative analyses will be conducted using SPSS Statistics (IBM Corp). Rapid qualitative analysis will be conducted using structured matrix templates and audit trails maintained in Microsoft Excel and Word; no dedicated qualitative software will be used.

Brief postintervention interviews will be analyzed using rapid qualitative content analysis with structured summaries and matrix displays to identify participants’ perceptions of the intervention, barriers and facilitators to implementation, and recommendations for refinement [53,54]. Quantitative and qualitative data will be analyzed first separately and then integrated using joint displays within a convergent mixed methods design. Quantitative outcomes (PAR, hearing protection beliefs, and implementation outcomes) will be compared with qualitative findings from the phase 2 postintervention interviews to examine how participants’ experiences help explain observed changes in hearing protector fit, hearing protection beliefs, and implementation outcomes. This integrated analysis will also identify opportunities to further refine the intervention prior to a future fully powered trial.

### Data Management and Security

Deidentified data will be stored on encrypted university servers with role-based access; REDCap (Vanderbilt University) Cloud (v15) will be used for electronic data capture. Version control for instruments, protocols, and analysis scripts will be maintained with regular backups and audit logs.

### Ethical Considerations

This study was reviewed and approved by the San Diego State University Institutional Review Board (protocol IRB-25‐0158). Verbal and written consent will be obtained. Participants in phase 1 will receive a US $40 gift card for completing formative interviews. Participants in phase 2 will receive a US $50 gift card for completing pilot study activities, reflecting the additional time required for intervention participation and outcome assessments. Risks are minimal and include potential temporary discomfort during earplug insertion.

## Results

Funding for this study was awarded in July 2025 through the San Diego State University’s HealthLINK Center for Transdisciplinary Health Research. Institutional Review Board approval was obtained from San Diego State University on April 28, 2025, and the study was registered on ClinicalTrials.gov (NCT07271290; first submitted on November 27, 2025, and first posted on December 9, 2025) prior to participant enrollment.

At the time of protocol submission, study materials, data collection procedures, and community-partner training activities had been completed. Recruitment for phase 1 formative interviews with farmworkers and supervisors was underway and expected to be completed by spring 2026. Analysis of phase 1 data is expected to continue through fall 2026, followed by adaptation of intervention materials and preparation for pilot testing. Recruitment for phase 2 pilot participants is anticipated to begin in July 2026, with completion of data collection expected in 2027. Data analysis and dissemination activities are planned for 2027. No substantive deviations from the approved protocol have occurred at the time of submission (July 2026).

## Discussion

### Principal Findings

This protocol describes a community-engaged Hybrid Type 1 pilot study designed to adapt and evaluate *Más Vale Usar Protección Auditiva*, a hearing loss prevention intervention for Spanish-speaking farmworkers. The study combines objective assessment of hearing protector fit through PAR testing with implementation outcomes and systematic adaptation procedures guided by CFIR and FRAME. By integrating CBPR principles with implementation science frameworks, the study seeks to generate evidence regarding the feasibility, acceptability, and preliminary effectiveness of a hearing conservation intervention tailored to an underserved agricultural workforce. We hypothesize that the adapted intervention will be feasible, acceptable, and appropriate for implementation in agricultural settings and will demonstrate preliminary improvements in objective hearing protector fit and hearing protection beliefs.

### Comparison to Prior Work

Previous hearing conservation interventions in agricultural populations have primarily focused on knowledge, attitudes, and intentions and have often relied on self-reported hearing protection behaviors as outcomes. Few studies have incorporated objective measures of hearing protector use or fit, and even fewer have focused specifically on Spanish-speaking farmworkers [16,18,28,56]. In addition, prior interventions have rarely incorporated implementation science frameworks or community-engaged adaptation procedures [28]. This study extends prior work by adapting an existing Spanish-language hearing conservation intervention for the agricultural context of the Southwest United States and by combining objective fit-testing, implementation outcomes, and systematic documentation of intervention adaptations. These features may improve the relevance, scalability, and reproducibility of future hearing conservation interventions in agricultural settings.

### Strengths and Limitations

A single-group pre-post design and small sample size limit causal inference and generalizability. Outcomes are assessed immediately following the intervention, and observed improvements in hearing protector fit or beliefs may partially reflect practice effects associated with repeated PAR testing, standardized earplug-fitting coaching, increased familiarity with the testing procedures, social desirability, or increased attention associated with study participation (ie, the Hawthorne effect). Accordingly, this pilot is designed to evaluate immediate changes in hearing protector fit and hearing protection beliefs and is not intended to assess long-term retention, sustained hearing protection use, or prevention of NIHL. However, findings from this study will inform the next stage of research with longer follow-up periods. Additionally, because behavior change is constrained by access and workplace conditions, self-reported hearing protection behaviors are treated as contextual indicators rather than primary outcomes in this pilot.

### Future Directions

This study will deliver the first culturally adapted, evidence-informed hearing conservation training evaluated with objective fit-testing for Spanish-speaking farmworkers in the Southwest United States. Results will establish feasibility and preliminary effectiveness, generate a practical playbook for community-based delivery, and lay the groundwork for a subsequent multilevel trial integrating supervisor and organizational components. This study also demonstrates the application of implementation science frameworks in community-based agricultural settings.

### Dissemination

We will pursue a structured, multipronged dissemination strategy tailored to community and scientific audiences. Community-facing outputs will include a brief summarizing key findings and practical recommendations, distributed via partner networks and a safety-talk slide deck for on-site use by employee safety leads. Dissemination materials will be reviewed through established CBPR partnerships prior to broader distribution.

### Conclusion

This protocol describes a community-engaged Hybrid Type 1 pilot to adapt and evaluate a brief, digitally delivered hearing protection training for Spanish-speaking farmworkers. The study will produce adapted training and facilitation materials addressing NIHL in agricultural settings, with systematic documentation of adaptations using FRAME, as well as detailed intervention reporting to support replication. By pairing objective fit testing (PAR) with pragmatic implementation outcomes (acceptability, feasibility, appropriateness), the pilot will assess whether this delivery approach is workable in real-world community contexts and will provide preliminary effect size estimates and operational guidance to inform a subsequent trial, integrating interpersonal and organizational components in a larger multilevel intervention. By prioritizing Spanish-speaking farmworkers (the predominant language group in the US agricultural workforce), this study targets a preventable occupational health burden while addressing persistent gaps in linguistically accessible hearing loss prevention.

## Supplementary material

10.2196/93134 Multimedia Appendix 1 Interview guide.

10.2196/93134 Checklist 1 TIDiER checklist for intervention reporting.

10.2196/93134 Checklist 2 StaRI checklist for author completion.

## Abbreviations

CAB: Community Advisory Board
CBPR: Community-Based Participatory Research
CFIR: Consolidated Framework for Implementation Research
FRAME: Framework for Reporting Adaptations and Modifications to Evidence-Based Interventions
NIHL: noise-induced hearing loss
PAR: Personal Attenuation Rating
StaRI: Standards for Reporting Implementation Studies
TIDieR: Template for Intervention Description and Replication
