# Supplementary material for: Hearing Loss Prevention Training for Spanish-Speaking Farmworkers: Protocol for a Community-Engaged Hybrid Type 1 Pilot Study
Source: JMIR Res Protoc. 2026 Oct 5;15:e93134. doi: 10.2196/93134 (PMC13638070; doi:10.2196/93134)
Supplement: Supplementary file 3 [file resprot-v15-e93134-s001.docx]

**Questions for Managers/Supervisors**

**Context and Role**

1. What is your job as a manager?
   - What are your responsibilities in the field?
2. How do people at your work usually think about safety rules?
   - Do they follow them carefully or more informally?
3. Do workers feel that protecting their hearing is part of being a good worker in this field?
   - Why or why not?

**Current Practices**

4. Do you provide your workers with hearing protection (earplugs or earmuffs)?

Tell me more...where, when, and how do you give them out?

5. If yes, in what area do the workers who receive hearing protection work?

6. What barriers exist in your workplace that prevent workers from using hearing protection, even when it is available?

7. Do you know of examples from other fields or jobs where workers are protected from noise?

**Reactions to the Video**

8. Have you ever shown a video like this to your workers?

9. Do you think this video could help your workers?

How would it help them? Or why wouldn't it help them?

10. Was there anything in the video that might cause confusion or not apply to your work?

What part was confusing or not applicable?

11. How well does the video reflect the real conditions at your workplace?

12. What parts of the video could be improved to make it clearer or more useful?

13. How useful do you think this video would be for motivating your team to use hearing protection?

14. What would make it more effective for them?

15. What information do you think was missing from the video?

16. How well could your workers learn to use earplugs just by watching the video?

**Implementation and Delivery**

17. Besides the video, what other things would help workers use hearing protection?

18. What changes would be necessary for hearing protection use to become a normal practice in your field

19. How could this video become a regular part of safety training in the field?

20. What factors (such as money, time, or rules) influence how this video can be shown?

21. How would you like to show this video to your team (for example, in a meeting, in the field, by WhatsApp)?

22. When would be the best time to share the video with workers?

23. Who at work would be the best person to encourage others to use hearing protection?

24. After presenting a video like this, how could we tell if it helped your team?

**Questions for farmworkers**

**Reactions to the Video**

1.     Have you ever used hearing protection before?

2.     Did the video show you a different way to put in earplugs, compared to how you usually do it?

3.     Did you learn anything (else) new about hearing protection from the video?

a)     What did you learn?

4.     What part of the video did you find most interesting?

a)     Why did it catch your attention?

5.     Was there anything you didn't understand well or that confused you?

a)     What was it that made you unsure?

6.     What situations in the video are similar to what you have seen at work?

7.     What example do you think was missing from the video?

8.     What would you change in the video to make it easier to understand?

a)     Could you explain why?

9.     What part of the video do you think was the most useful?

a)     Could you explain why?

10.     What part did you feel was not so important?

a)     Could you explain why?

**Social Norms**

11.     How do you think your coworkers would react to watching this video?

12.     What parts of the video might motivate other workers to use hearing protection?

13.     What would you say to a coworker who refuses to use hearing protection?

14.     If we wanted to share this video with you (the workers), what would be easiest?

a)     If we sent it by WhatsApp?

b)     If you watched it together at work, during breaks, or in a meeting?

c)     Or is there another way you prefer?

**Barriers and Facilitators**

15.     What things make it hard to use earplugs or earmuffs?

a)     For example: they hurt, there isn't enough time, the supervisor doesn't give them out, they fall out, etc.

16.     What would help or motivate you to use earplugs or earmuffs?

a)     For example: being shown how to put them in, being comfortable, always having them available and free.

17.     What do you think about the cost of hearing protection (earplugs, earmuffs)?

18.     Do you think the price affects whether people use them or not?

a)     Why?

19.     What do you need to make it easier to protect your hearing at work?

**Preferences**

20.     When do you think would be a good time to watch this video?

a)     For example: before work, in a meeting, or another time?

21.     How would you prefer to learn step by step how to put in the earplugs?

a)     For example: watching a live demonstration, practicing with the earplugs yourself, or only with the video.
